# Pattern Completion and Rate Remapping in Retrosplenial Cortex

**DOI:** 10.21203/rs.3.rs-2736384/v1

**Published:** 2023-04-14

**Authors:** Zaneta Navratilova, Dhruba Banerjee, Fjolla Muqolli, Jordan Zhang, Sunil Gandhi, Bruce McNaughton

**Affiliations:** University of California-Irvine; University of California-Irvine; University of California-Irvine; University of California-Irvine; University of California-Irvine; University of Lethbridge

**Keywords:** Hippocampal-cortical interactions, Place cells, CA1, Global remapping, Memory index theory

## Abstract

Principles governing the encoding, storage, and updating of memories in cortical networks are poorly understood. In retrosplenial cortex (RSC), cells respond to the animal’s position as it navigates a real or virtual (VR) linear track. Position correlated cells (PCCs) in RSC require an intact hippocampus to form. To examine whether PCCs undergo pattern completion and remapping like hippocampal cells, neuronal activity in RSC or CA1 was recorded using two-photon calcium imaging in mice running on VR tracks. RSC and CA1 PCC activity underwent global and rate remapping depending on the degree of change to familiar environments. The formation of position correlated fields in both regions required stability across laps; however, once formed, PCCs became robust to object destabilization, indicating pattern completion of the previously formed memory. Thus, memory and remapping properties were conserved between RSC and CA1, suggesting that these functional properties are transmitted to cortex to support memory functions.

## Introduction

The retrosplenial cortex (RSC) has been implicated in memory processing through loss-of-function, anatomical, and functional imaging studies. Patients with brain damage localized to RSC experience anterograde and retrograde amnesia ^1-3^, while excitotoxic lesions or inactivation of the region in experimental animals disrupt learning in spatial navigation tasks and recall of associations ^4-7^. Anatomical tracing studies across species show that RSC selectively receives inputs from other regions associated with memory, including the hippocampus and entorhinal cortex ^8,9^. Additionally, RSC becomes functionally engaged during memory tasks in humans ^10^. These studies raise questions as to the nature and dynamics of information coding and storage in RSC.

At the cellular level, activity patterns in RSC show notable similarities to those in other memory-associated brain regions. The most striking, and first discovered, pattern of activity in the temporal lobe is the highly localized firing of “place cells” in the hippocampus ^11^. In recent years, recordings have been made from RSC neurons in live animals navigating through real or virtual reality (VR) environments. In mice running through a linear corridor, position-correlated cells (PCCs) were found within the RSC^12-14^. Each PCC fires when the animal enters one or a few specific portions of the track, known as that cell’s place fields, such that when the mouse runs the length of the corridor, the corresponding PCCs become sequentially active. Cortical PCCs resemble^13,15-17^ and fluctuate coherently with hippocampal CA1 place cell sequences ^18^. They are also driven by cues similar to those that drive hippocampal place cells ^19-21^ and depend on an intact hippocampus to form ^14,22^. Once established, however, RSC position-correlated responses survive hippocampal lesions ^23^.

Hippocampal place cell representations are hypothesized to support memory by creating a spatiotemporal framework in which cortically represented experiences can be embedded ^24,25^. Changes, or remapping ^26,27^, of place cell representations exhibit many properties expected from a network involved in memory processing. Major alterations to the environment induce global remapping, where hippocampal activity in one spatial context does not predict activity in another^28^. A sparse subset of cells is allocated to represent each context, with overlap close to chance levels, increasing storage capacity while avoiding interference ^29-31^. A distinct representation develops within minutes of exposure to a new context, with many fields forming on the very first exposure ^26,32-35^. Therefore, remapping enables the hippocampus to rapidly incorporate new information into associative circuits while minimizing interference with older memories^36^. Hippocampal representations are not easily perturbed from an established activity pattern, however. Just as a memory network tends to gravitate towards particular activity states ^31,37-39^, hippocampal activity persists with the same representation for a variety of similar inputs ^40,41^. This pattern completion enables the recall of memories from noisy or incomplete sensory cues. Changes from a stable hippocampal representation that do eventually occur lag the changes in external input^41^, showing how prior experiences modulate the influence of external input. Finally, hippocampal place cell representations extend their capacity to encode experiences by exhibiting multiple encoding schemes. In contrast to global remapping, when multiple events occur within the same spatial context, the place cells’ fields are maintained, but the cells’ peak firing rates change ^42^. This has been termed “rate remapping,” and is thought to provide a mechanism whereby hippocampus rotates its output vector for different experiences at a given location without changing the basis set for that position ^43^.

In summary, remapping properties of place cell representations have revealed a great deal about how the hippocampus processes memories. In contrast, little is known about cortical PCC remapping. In this study, we investigated remapping properties of the dysgranular portion of RSC and hippocampal CA1 using two-photon imaging in mice running while head fixed and engaging with visual VR environments.

## Results

### 2P Imaging in RSC and CA1 during VR experience

**Figure 1:**
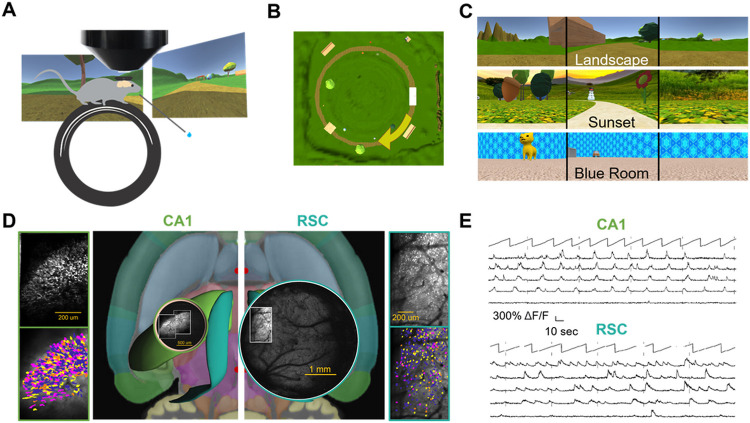
Imaging RSC and CA1 neurons while mice run in virtual reality environments. **A.** Each mouse was head-fixed over a running wheel, with tablets displaying a VR environment surrounding the mouse from three sides. Cartoon image illustrates the mouse with two tablets (left and front; third tablet, right side, not shown). A reward spout dispensed water or milk rewards. A wide-angled mesoscope recorded neuronal activity above the craniotomy. **B**. Aerial view of the circular VR track environment called “Landscape.” Each lap starts inside the tunnel (white rectangle on the right side of the image) and proceeds clockwise (in the direction of the yellow arrow, which is overlaid on the aerial view). **C**. Screenshots from three example environments showing views on the left, front, and right tablets. **D**. Transverse mouse brain diagram showing a 3D rendering of retrosplenial cortex (RSC) in teal, and hippocampal CA1 in lime (center; Allen atlas). Overlaid are panoramic two-photon images acquired over either hippocampal CA1 (left circle) or dorsal neocortex (right circle) in separate mice. Regions of interest overlying hippocampal CA1 or RSC were selected within the panoramic view and recorded at 6-8 Hz. Max projection of example imaging sessions over CA1 (top, left) and RSC (top, right) are shown. Bottom right and left show cells detected by Suite2P and manually curated. Neurons are color-coded based on whether they had significant spatial tuning in the familiar (yellow), novel (pink), both (orange), or neither (blue) environments. **E**. ΔF∕F activity traces from five example cells in CA1 (top) and RSC (bottom) are shown. Position along the track as a function of time for each session is shown above the activity traces.

To study PCC sequences, we trained mice to run on a simple visual VR task. Mice were head-fixed above a wheel, and viewed visual stimuli on three surrounding tablets (Fig. 1A). Self-induced rotation of the wheel instantaneously moved the animal around a VR circular track environment. To motivate running, mice were presented milk or water rewards at hidden reward sites along the track. After 4-6 weeks of training in a single environment (familiar), most mice learned to comfortably run over 20 laps in each session.

To study responses in novel environments, several visually distinctive VR environments were created. All were 3-5 meter circular tracks with a common 30 cm ‘tunnel’ cue at the start, and various other visual objects on either side of the track. The novel track differed from the familiar in circumference, background images, objects along the track, location of (two) reward sites, and sometimes the direction of the curve of the track (clockwise vs. counterclockwise) (Fig. 1B&C). After the animal had been habituated to running in the familiar environment while undergoing imaging (2-5 days), the experimental session commenced. Pre-task rest activity (with the wheel blocked from movement) was imaged for 10-30 min before and after each running session, to detect all neurons with any spontaneous activity. Afterwards, the animal was allowed to run for approximately ten minutes in the familiar environment (at least 10 laps). Once back at the ‘tunnel’, the mouse was instantly teleported into the novel environment, where it continued running for 15 or more laps. To quantify behavioral memory, we measured in which position bins mice licked on each lap. The five (out of 100) bins just prior to each reward delivery were considered “correct,” and the 5 bins following reward delivery were excluded (see Methods). Mice licked more accurately (in proportion of licks pre-reward) in the familiar than in the novel environment (0.400+/−0.177 vs. 0.270+/−0.190, paired t-test, p<0.05). In both environments, mice licked in the prereward zone more often than chance (0.111; familiar t-test, p<0.001; novel t-test, p<0.001). Once in the novel environment, licks at the former, familiar environment reward locations were no more than at chance level (0.106+/−0.063, t-test, p=0.66). There was no difference in lick performance between mice that were imaged in RSC and mice that were imaged in CA1 (two-way ANOVA, effect of brain region, p=0.87).

Two-photon calcium imaging was used to record cellular-resolution neural activity in RSC or hippocampal CA1 as animals performed the task. In one group of transgenic mice (CaMK2a-tTa x tetO-GCaMP6s or Thy1-GCaMP6s), layer 2/3 excitatory cells were imaged from dysgranular RSC through a glass coverslip (11,232 cells from 18 sessions in 16 mice). In the second group of transgenic mice (Thy1-GCaMP6s only), pyramidal cells in CA1 (7,357 cells from 10 sessions in 6 mice) were recorded through a 1.8mm cylindrical micro-optic plastic inserted into a volume of aspirated somatosensory cortex (Supplementary Table 1 shows a list of all analyzed datasets for experiment one). Recordings yielded hundreds of cells (with at least 1 calcium spike) per session (Fig 1D), from which time-varying calcium signals were extracted (Fig. 1E), aligned to VR and behavioral data, and position binned (see Methods for details).

#### PCCs in familiar and novel environments

**Figure 2:**
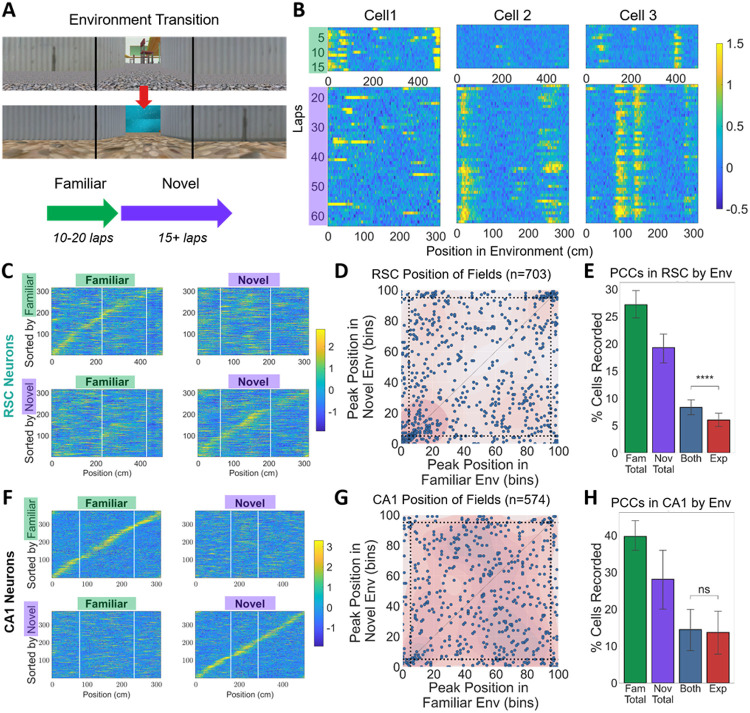
RSC cells exhibit hallmark features of global remapping in novel VR environments. **A.** Each mouse was trained for 3+ weeks in one of the environments (familiar), and then imaged during a session in which it ran 10-20 laps in the familiar environment and then was teleported to a visually distinct novel environment for 20 min (15+ laps). The schematic shows this teleportation protocol. **B.** Z-scored position binned activity of example RSC cells across laps of the familiar (top) and novel (bottom) environments. Cell 1 established a place field only in the familiar environment, cell 2 exhibited two place fields in the novel environment and none in the familiar, while cell 3 had fields in both the familiar and novel environments but at different relative spatial separations. **C.** Representative data from all RSC cells imaged simultaneously in a single session. Left column shows z-scored, lap-averaged activity in a familiar environment. The right column shows the activity of the same cells averaged across laps in a novel environment. Cells are ordered by the position on the track at which they exhibited peak activity in the familiar environment in the top row, and the novel environment in the bottom row. Activity from half of the final ten laps was removed before averaging and used to determine the order of peak activity. **D.** For cells in all sessions that had a single field in both environments, the position of peak activity in the familiar versus novel environments is plotted. Dotted lines indicate the extent of the 30 cm virtual tunnel. The background shading indicates the kernel density estimate for the scatterplot. **E.** The proportion of RSC cells with spatial tuning were tabulated for the familiar (green), novel (purple), and in both environments (grey-blue). The proportion of cells with fields in both environments was compared to the expected value from a random process with replacement (Exp, red). **F. G. H.** Same as D. E. F. but for cells imaged in CA1. Error bars represent SEM.

In experiment one, we examined position correlated activity in familiar and novel environments (Fig. 2A). To visualize activity in each session, we separated the data by environment and plotted the lap-by-lap activity of each cell as a function of position (Fig. 2B). To classify cells carrying position information, a multi-step criterion was applied to the calcium signal from cells in both RSC and CA1 (see Methods for details). In laps of the familiar environment, on average 27.2% (+/− 10.6) of RSC and 39.7% (+/− 12.8) of CA1 cells recorded exhibited position-related activity (and thus were classified as PCCs). The average spatial information content (SI) across all cells was calculated to complement this criterion-based approach. The SI was slightly higher in CA1 compared to RSC cells (0.83 bits per second (bps) +/− 0.08 vs 0.71 +/− 0.11, t=3.05, p=0.005). During the novel environment laps, a substantial number of cells passed the PCC criteria, 19.3% (+/− 11.4) in RSC and 28.1% (+/− 25.9) in CA1. The average SI was significantly lower in the novel environment than in the familiar, for both RSC (t=3.07, p=0.004) and CA1 (t=3.03, p=0.007), and again significantly different between areas (0.73+/−0.07 bps in CA1 and 0.61+/−0.09 in RSC, t=3.59, p=0.001). Therefore, although position correlated activity was highest when animals ran in familiar environments, a still sizable number of PCCs (71% of the familiar environment count) could be identified in both RSC and CA1 on the first day in a novel environment.

### Novel environments caused global remapping of RSC activity

Next, to characterize PCC remapping, we examined how the neural activity differed across environments. Cells were ordered based on the position at which their lap-averaged activity peaked in each environment. Plotting in this manner revealed pronounced sequences in RSC and CA1 in both the familiar and novel environments (example in Fig. 2C&F top left and bottom right, respectively).

We looked for hallmarks of global remapping by comparing changes in cell-specific neural activity in the familiar and novel environments. To evaluate orthogonality, we applied the order in which cells were sorted in one environment to the lap-averaged activity in the other environment. Rearranging the cells in this way showed that the sequence was not preserved across environments (example in Fig. 2C&F bottom left and top right, respectively). These results were consistent across both regions and all animals tested, suggesting that sequences in one environment are unrelated to sequences in another. The hippocampal results confirm that the current VR paradigm can replicate long-standing global remapping results from real-world experiments, while RSC results demonstrate that RSC cells’ activity changes in a manner consistent with global remapping.

Global remapping requires that even cells active in multiple environments would have uncorrelated activity patterns. For cells that had a single field in both environments, the locations of those fields across environments were uncorrelated in CA1 (r=0.01; Fig. 2G; Table 1). In RSC, there was a significant correlation between field locations (r=0.24; Fig. 2D; Table 1). The relation was driven primarily by cells with fields near the common ‘tunnel’, however, excluding just those fields (within five bins of the beginning or end of the track, marked by dotted lines) left no significant correlation between remaining field locations in RSC cells (r=0.09). Thus, for most of the track, there was no relationship between where the cell would establish fields in the novel and familiar environments.

Efficient pattern separation also requires that each environment be encoded by an uncorrelated subset of cells. If position-correlated fields are allocated randomly and all imaged cells are equally likely (this is known to be false: pyramidal cells have different excitabilities, and thus different likelihoods of activation/allocation ^44,45^; but in small environments, this assumption can be helpful; see also Fig S1), the expected proportion of cells with position-correlated fields in both environments should be the proportions of PCCs in each of the environments, multiplied together. Indeed, we found that the real proportion of cells with fields in both environments was close to the mathematically expected value. The real proportion of RSC was significantly but weakly higher than expected (2.32% higher; Fig. 2E; Table 1). Removing cells with fields near the tunnel reduced the RSC difference from expected (1.70% higher) but it remained significant. Thus, in RSC, cells with fields in one environment were only slightly favored to have a field in another. The real proportion of CA1 cells was not significantly different from expected (0.76% higher; Fig. 2H; Table 1).

### Population activity changed immediately upon entry into the new environment and then evolved into a new stable representation

**Figure 3:**
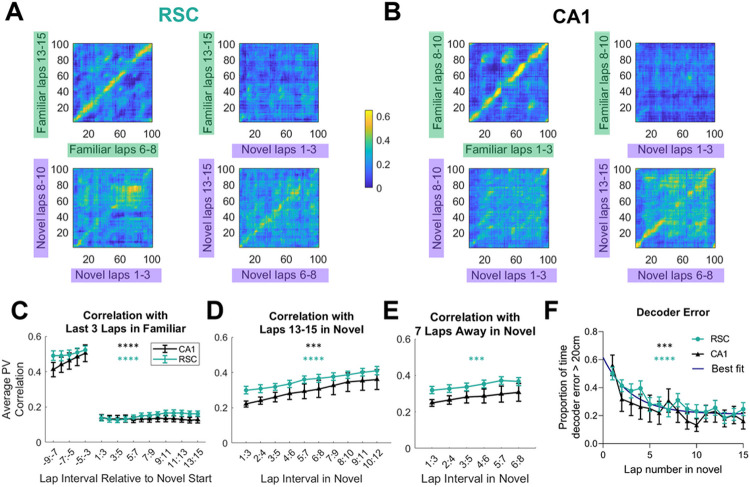
Population vectors show an abrupt change at the switch in environments, and then a gradual stabilization of population activity in the novel environment. **A.** The activity of all simultaneously imaged RSC neurons during an example session in each position bin (the PV), averaged across a 3-lap interval, is correlated with all position bins during a different 3-lap interval. Top row shows the correlation of late familiar environment laps with early familiar environment laps (left) and late novel environment laps (right). Bottom row shows the correlation of late novel environment laps with early novel environment laps (left), and middle novel environment laps (right). **B.** Same as A except using CA1 cells imaged in a different mouse. **C-E**. The PV for each 3-lap interval was correlated against the same spatial bins in a reference set of laps. The average across bins was taken as the correlation for each set of laps (i.e. averaging along the diagonal in the correlation matrices shown in A). **C.** The reference laps are the final three laps in the familiar environment, and were correlated with every non-overlapping lap interval before and after the transition to the novel environment. **D**. The correlation between late laps (13-15) in the novel environment to all non-overlapping earlier lap intervals. **E**. Each lap interval was correlated with an interval 7 laps later (e.g. laps 1-3 with laps 8-10, and laps 6-8 with laps 13-15). **F**. The proportion of time in each novel environment lap for which a Bayesian decoder trained on all other novel laps was off by more than 20 cm. Error bars represent SEM. Asterisks indicate that repeated measures ANOVA showed a significant effect of laps for RSC (teal), or CA1 (black) datasets.

The substantial fraction of PCCs in the novel environment suggests that both regions could form a representation of the environment within several minutes. To understand the dynamics of PCC sequence formation, we first examined how the activity changed in the population. We calculated a population vector (PV) at each position bin of the environment, averaged over a sliding window of three consecutive laps. These PVs gave the state of the neural network at a specific bin for a specific lap interval. The PVs at the final (or late) laps in each environment were then correlated against PVs in other lap intervals. Early laps in the familiar environment were highly correlated to final laps, suggesting that the network rapidly entered a stable state when presented with well-trained inputs (example in Fig. 3A&B top left). As expected from the global remapping findings, familiar laps showed no correlation to novel laps (Fig 3A&B top right).

PV correlations between each bin and the corresponding bin in a different set of laps were averaged (i.e. along the diagonal in Fig 3A&B). Correlating the last 3 laps in the familiar environment with all other non-overlapping sets of laps showed that remapping occurred immediately upon entry into the novel environment (Fig 3C; Table 1). Correlating late laps (13-15) in the novel environment with previous novel environment laps showed a gradual rise in similarity (Fig 3D; Table 1). To check if this steady rise was due to gradual drift across laps, or to an increasing stabilization of population representations, each lap interval was compared to an interval 7 laps later (Fig 3E; Table 1). RSC still showed a significant increase in stability across laps. CA1 datasets showed a lot of variability, and thus we were not able to show a significant effect of laps, even though some datasets clearly showed stabilization of representations (Fig. 3B). In sum, these data show that, in a novel environment, RSC population activity immediately de-correlates from that in the familiar environment, and then a new representation stabilizes across subsequent laps.

To further determine the stability of the neural representations of novel environments, we used a Bayesian decoder to predict the position of the mouse from RSC or CA1 activity in each lap, using a decoder trained on every other lap (leave one out cross validation). There was no difference in the mean decoder error between RSC and CA1 in either familiar (RSC: 9.7 +/− 6.8 cm; CA1: 9.6 +/− 5.1 cm) or novel (RSC: 30.6 +/− 14.9 cm; CA1: 26.2 +/− 16.4 cm) environments. The decoder error was higher in the novel environment compared to the familiar environment, even when considering only the best 5 laps of the session (RSC: familiar 5.2 +/− 2.3 cm; novel 14.2 +/− 12.8 cm; CA1: familiar 3.8 +/− 1.2; novel 11.2 +/− 10.5, Table 1). To determine if the decoder accuracy increased across laps, we calculated for how long proportionally the decoder error was greater than 20 cm (3-6 position bins) during each lap (Fig 3F). This proportion decreased significantly across laps in both CA1 and RSC datasets (Table 1). There was again no difference between brain regions.

### In-field activity increased during novel field formation

**Figure 4:**
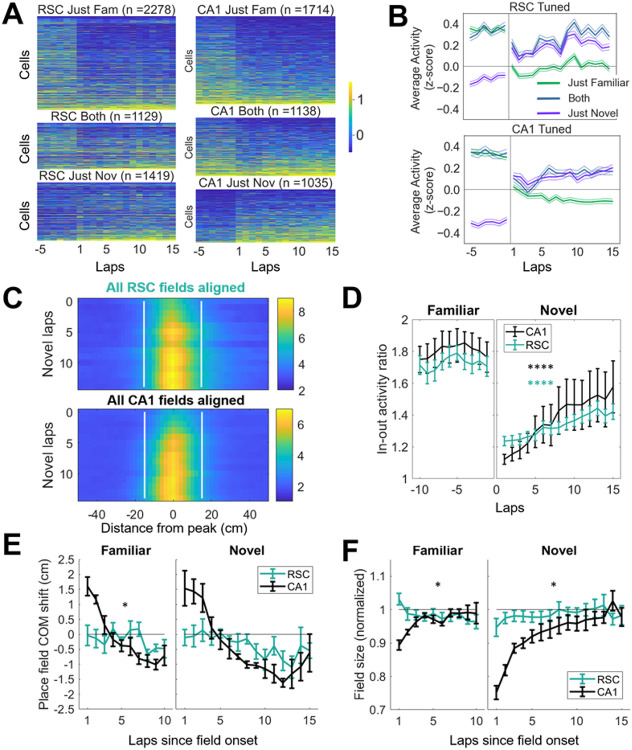
Mean activity and activity within position fields tunes up over the course of a few laps in each environment. **A**. Lap averaged Z-scored calcium activity for all recorded cells spatially tuned to just the familiar (top), both (middle), and novel (bottom) environments (RSC left, CA1 right) for laps surrounding the environment transition. Cells are ordered by mean activity in the final laps in the novel environment (for most sessions this was later than lap 15). **B**. Average z-scored activity of all cells tuned to the familiar, novel, or both environments. **C**. To visualize the evolution of place fields in the novel environment, all RSC (top) and CA1 (bottom) place fields (only for cells that were classified as PCCs) across all novel day sessions were aligned to their peak bin, and their fluorescence activity across laps was averaged. **D**. A ratio was computed to quantify how activity within the field (a 30 cm region around the peak bin of each cell) evolved relative to activity outside the field **E**. The center of mass (COM) was calculated for each place field (each spatially-tuned cell could have one or more place fields) in the familiar and novel environments by weighting each position bin within the boundaries of the field with the mean activity in that bin. Then the COM was calculated for each lap with above threshold activity, and subtracted from the mean COM for that field. CA1 cells showed a COM shift similar to previously published data. RSC cells showed no significant shift. **F**. Field size was calculated for each place field by calculating the number of bins within place field boundaries that had above-threshold activity (>50^th^ percentile of all bins), and then normalized to the average across laps. Laps with mean activity below threshold were excluded from analysis, same as in E. CA1 fields increased in size across laps in both familiar and novel environments, while RSC cells did not. Asterisks indicate that repeated measures ANOVA showed a significant effect of laps for RSC (teal), or CA1 (black) datasets.

Next, we investigated the dynamics of PCCs and their position-correlated fields. To evaluate changes in average PCC firing rates across the session, we grouped PCCs across sessions according to whether they exhibited fields in just the familiar, just the novel, or both environments. Neural activity from these cells was averaged across each lap then z-scored by session (Fig. 4A&B). As expected, the group of PCCs with fields in just the familiar environment dropped in activity once the animals entered the novel environment, whereas activity rose in PCCs with fields in just the novel environment (Fig. 4B). Interestingly, the activity of PCCs with fields in both environments dropped by 69.2% in CA1 and 41.73% in RSC (see Table 1) upon entering the novel environment, as has been described previously in CA1.^46^

Next, we analyzed the activity within place fields, by aligning the activity of all cells to their peak activity bin. Plotting the average PCC field activity for each lap showed that in-field activity was low in the first several laps (Fig. 4C). We quantified this by calculating the ratio between in-field (within a 30 cm region surrounding the peak) and out-of-field (all other position bins) activity for all cells (not just PCCs; Fig 4D). The in-to-out field activity ratio increased over the course of the first several laps, for both RSC and CA1 (Table 1). There was a significant difference in the slope of the increase in in-to-out field ratio between brain regions (in-out ratio increased faster in CA1; Table 1). Taken together, these findings show that the gradual increase in population decoding of position information is accompanied by an increase in firing rates of PCCs with fields in the novel environment, and an increase in in-field firing rates. There was no difference between RSC and CA1 in most of these measures, suggesting that spatial information is shared between the two regions right from the beginning of a new memory.

### Experience-dependent asymmetric place field expansion does not occur in RSC

A major effect of repeated route following in the hippocampus is the phenomenon of backwards shift and expansion of place fields, which is thought to indicate asymmetric plasticity between neurons representing sequential positions, and is dependent on NMDA receptors ^47^. CA1 place cells show backwards shift and expansion, in both familiar and novel environments ^48^. To check if this phenomenon might also occur in RSC or be transmitted to RSC from hippocampus, we calculated the center of mass of each field on each lap (see Methods). Because fields were not expressed on every lap, especially in the novel environment, only laps with significant in-field activity, starting with the onset lap for each field, were considered. As in previous studies, CA1 PCC fields showed a backwards center of mass shift in the familiar environment (Fig. 4E left; Table 1). RSC PCCs showed no such shift (Fig. 4E left; Table 1). Given the large variability in spatial stability between CA1 datasets (see error bars in Fig 3 C&D), we focused only on the datasets with high spatial tuning in the novel environment (datasets with >12 % PCCs in the novel environment, which was 12/18 RSC datasets, and 5/10 CA1 datasets; see Supplementary Figure S1 for results using all datasets). In this subset of the data, CA1 fields in the novel environment showed a trend towards a backwards COM shift, while RSC fields did not (Fig 4E right, Table 1).

To confirm place field expansion, place field size was calculated across laps. For each lap (with significant activity), the number of bins within the average field boundaries that had above threshold ΔF∕F was counted. As with COM shift, CA1 fields showed field expansion in the familiar environment, but RSC fields did not (Fig 4F left; Table 1). In the novel environment, CA1 fields also showed place field expansion, and RSC fields did not (Fig 4F right; Table 1). In conclusion, although RSC place fields appeared and developed similarly to CA1 fields, they did not show the hallmarks of NMDA receptor-dependent sequential coupling that are observed in hippocampus.

### Shifting visual objects disrupts field formation

**Figure 5:**
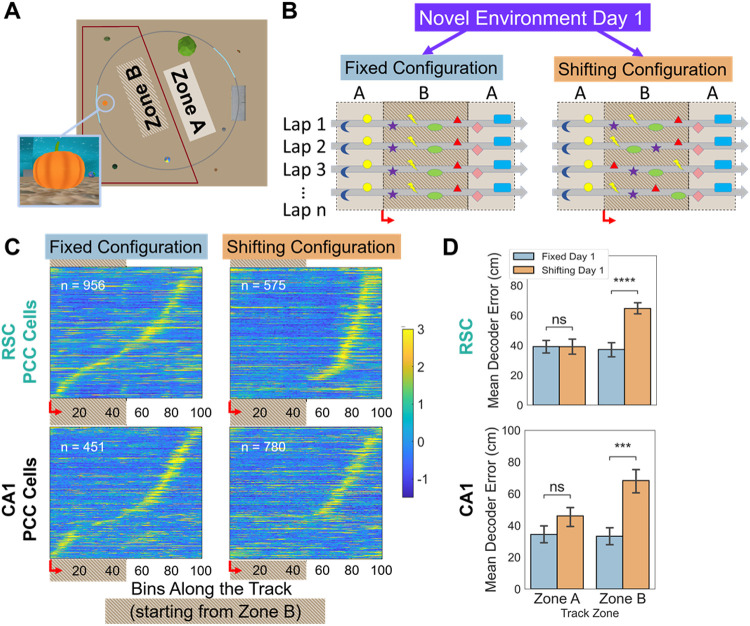
Shifting Objects Disrupt PCS Formation. **A.** Aerial view of a circular track VR environment, divided into zones A and B. Each zone contained 4 objects of various shapes and sizes, one of which is shown enlarged from mouse point-of view. **B.** Schematic of two environment configurations. Animals were introduced to a novel environment in the fixed or shifting configuration. In the shifting configuration, the 4 objects in zone B move to new positions on each lap. **C.**Activity of PCCs in zone B (bins 1-50) and zone A (bins 51-100) for either the fixed or shifting configurations. Activity in each session is z-scored and then cells are aggregated across sessions and arranged by their peak location. **D.** Mean Bayesian decoding error in zones A and B in the novel environment sessions, run in either the fixed (blue) or shifting (orange) configurations.

In the second experiment, we investigated whether a consistent visual experience was necessary for position correlated fields in RSC and CA1 to develop. Animals were introduced to VR environments with lap-to-lap variability. These environments consisted of a uniformly patterned background (e.g. “Blue Room” in Fig. 1C) and eight visually distinct objects distributed around a circular track. The track was split into two equally sized zones (“A” and “B”), each with landmarks occupying four of six possible locations and one reward site (Fig. 5A). In the fixed configuration, objects in both zones maintained the same location every lap. In the shifting configuration, objects in zone B randomly switched locations within the zone, on a lap-to-lap basis (Fig. 5B). Zone A acted as a negative control, with fixed objects in both conditions. Mice did not see the objects move, because the objects shifted while the animals were inside the opaque ‘tunnel’ in the middle of zone A. In this experiment, mice were divided into two groups. Both groups were first trained in a familiar environment, and then introduced to a novel one. One group experienced the novel environment in the fixed configuration, with no objects shifted at any time (n=10 RSC, 6 CA1 sessions). The second group experienced the novel environment with zone B objects shifted on every lap from the start, and thus never saw the same configuration of zone B objects twice (n=9 RSC, 7 CA1 sessions). Most mice participated in multiple experiments or groups, but a new novel environment was used for each experiment (see Supplementary table 2 for all experiments each mouse ran).

As with the prior experiment, when animals ran in the fixed configuration, position-correlated fields rapidly formed across the track, creating a PCC sequence in both RSC (Fig. 5C top left) and CA1 (Fig 5C bottom left). The same was true for zone A of the shifting configuration; however, few fields developed in zone B for RSC, and a clear PCC sequence was absent in both RSC and CA1 (Fig. 5C right; Table 1). RSC PCCs with fields in zone B had lower SI in the shifting compared to the fixed configuration. Interestingly, for CA1, this was true for PCCs in both zone A and zone B (Table 1). To verify this paucity of fields without a PCC-criteria based approach, we examined how well neural activity at the population level could predict the animal’s position using a Bayesian decoder. Factors significantly influencing decoding errors were statistically tested by constructing linear mixed-effects models, with mouse and session as random effects. We found there was no significant difference in zone A decoding errors between the fixed and shifting configurations for RSC animals or CA1 animals (Fig. 5D; Table 1). In zone B, however, decoding errors were twice as high in the shifting compared to the fixed configuration for both RSC and CA1 animals (Table 1). The selective absence of PCC sequences and poor population-level decoding near the shifting objects suggested that both CA1 and RSC require a consistent visual experience to form position correlated fields.

### Shifting visual objects does not disrupt field maintenance

**Figure 6:**
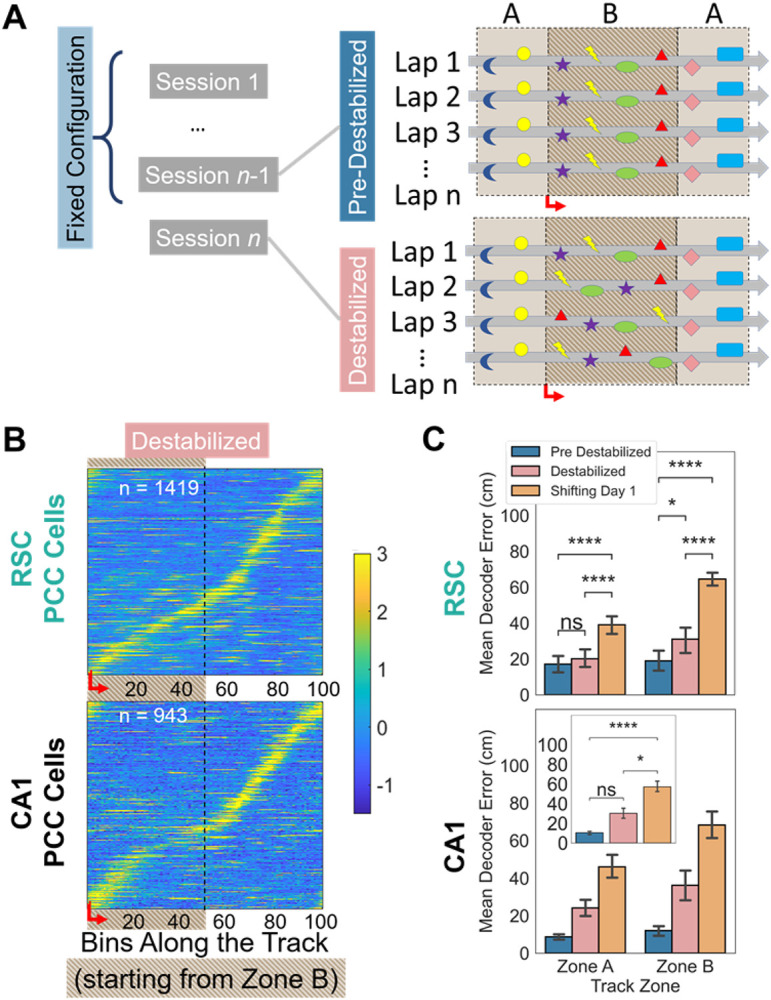
Once formed, PCC sequences resist disruption by destabilized objects. **A.** Schematic of the experimental protocol. Mice that were exposed to an environment in the fixed configuration for at least 3 days (sessions 1 through n-1) were then exposed to the same environment in a shifting configuration (session n). **B.**Activity in of PCCs (cells that passed criterion for spatial tuning) in zone B (bins 1-50) and zone A (bins 51-100) for the destabilized sessions. Activity in each session is z-scored and then cells are aggregated across sessions and arranged by their peak location. **C.** Mean Bayesian decoding error in zones A and B in the pre-destabilized (day n-1; blue), destabilized (day n; pink) configuration sessions, or in a novel environment in shifting configuration (different environments and/or mice; same data as Fig. 5D). There was no significant difference between zones in CA1, and so the combined differences between configurations are shown in the inset.

In the third experiment, we investigated whether a consistent visual experience remained necessary to maintain established PCC sequences in a familiarized environment. For three or more sessions, animals ran in an environment in the fixed configuration (all eight visual objects maintained the same locations). After these familiarization sessions, animals experienced the same environment (same background, visual objects, and reward locations) in a destabilized configuration, in which visual objects in zone B began shifting to new locations within the zone every lap (Fig. 6A). The lap-to-lap variability of the destabilized configuration was identical to the first day in the shifting configuration in experiment two, except that animals in the former had previously had an opportunity to form a memory of the environment in the fixed configuration. The transition to destabilization occurred after 10 or more laps either in a different (highly) familiar environment (n=2 RSC, 3 CA1 imaging sessions) or in the fixed configuration of the same environment (n=4 RSC, 1 CA1 imaging sessions); in either case the preceding laps/session with fixed objects in the same environment were known as the pre-destabilized configuration. To ensure the environment had been adequately learned prior to destabilization, mice with pre-destabilized average decoding errors >30 cm were excluded (2/8 RSC and 1/5 CA1 sessions). We compared position correlated activity in RSC and CA1 cells from animals experiencing the pre-destabilized, destabilized, and shifting configurations.

As expected, mice exhibited prominent position-correlated fields across the track in the pre-destabilized session. A sizable percentage of all cells in each session exhibited fields (25.6+/−17.2% for RSC sessions, 34.5+/−15.7% of which were in zone B; 40.0+/−9.2% for CA1, 38.7+/−8.9% of which were in zone B), and decoding accuracy was high in zone B (Table 1). When these mice transitioned into the destabilized configuration, distinct position-correlated fields remained present in zone B for both brain regions (24.8+/−18.7% in RSC, 27.5+/−20.5% of which were in zone B; 32.4+/−9.2 in CA1, 32.7% of which were in zone B; Fig. 6B; Table 1). In RSC, the decoding error in predicting the animal’s position from destabilized configuration activity was only slightly higher than pre-destabilized but about half as low as the first day in shifting (Fig. 6C; Table 1). Decoding errors were higher in zone B and differences between object configurations were more pronounced in zone B than A. In CA1, there was a significant difference in average decoding error between shifting and both the pre-destabilized and destabilized configurations. Interestingly, zone was not a significant factor in CA1, indicating that the effect of visual object configuration extended across the entire track. Therefore, although animals had the same single day experience with inconsistent visual objects in the shifting and destabilized configurations, in the latter case the animal’s memory of the environment enabled RSC and CA1 to maintain position-correlated fields and retain population-level position information.

### RSC cells do not track shifting visual objects

**Figure 7:**
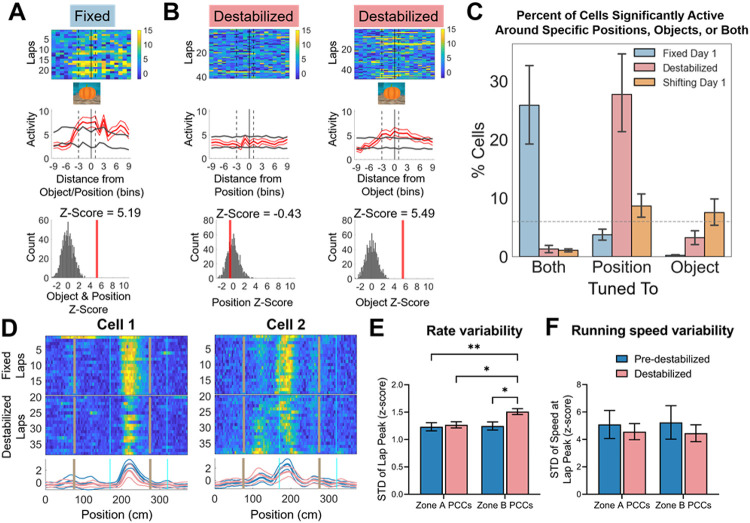
Negligible Proportion of RSC Cells Track Shifting Visual Objects, but RSC PCCs show Rate Remapping **A.** Activity of an example RSC cell in the fixed configuration, aligned to the position where the animal passes the pumpkin object. Activity on individual laps (top), averaged with standard error lines for all laps in the fixed configuration (middle red). Thick gray lines indicate 2.5^th^-97.5^th^ percentile from a 1000x shuffled distribution of cell activity. Activity within the dashed vertical black lines was considered close to the aligned position/object. A cell was considered tuned if: 1) lap-averaged cell activity minus standard error exceeded the 97.5^th^ percentile shuffle at any point close to the aligned object/position, and 2) activity averaged in all bins close to the aligned object/position exceeded a z-score of 2.33 (p<0.01) relative to the activity in those bins in the shuffled distribution (bottom). In the fixed configuration, this cell was considered significantly tuned to both position and object. **B.** Same cell as A on the same day, but now the environment is in the destabilized configuration. Left, activity aligned to the position where the pumpkin had previously consistently appeared. Cell is no longer significantly tuned to position. Right, activity aligned to whichever bin where the pumpkin object appears. Cell continues to be significantly, but not consistently tuned to object. **C.** Percentage of recorded cells tuned to position, object, or both, separated by configuration. Dashed line indicates the expected false positive rate for the criteria. **D.** Position binned activity of two example RSC cells with place fields in zone B, as a function of position on the track and lap number. Laps 1-19 were in the fixed configuration. Laps 20-38 show activity in the destabilized configuration. Region contained between thick vertical brown lines represents zone B, thin vertical cyan lines indicate reward locations. Bottom, lap-averaged, smoothed activity from fixed (blue) and destabilized (pink) configurations were plotted together as a function of position on the track. Thick lines indicate lap-mean; thinner lines indicate standard deviation. **E.** Standard deviation across laps of peak activity of spatially tuned RSC cells in the pre-destabilized session, and in the destabilized session. Pre-destabilized (blue) or destabilized (pink) session averaged values were compared for cells with fields in each zone. Destabilization resulted in a significant increase in rate variance, consistent with rate-remapping. **F.** Standard deviation across laps of the running speed at which mice passed through the peak of each PCC’s field. There was no difference in running speed variability between zones or configurations.

We considered that the absence of fields in the shifting configuration may be the result of cells responding to individual visual objects, instead of position along the track (i.e. the distance from the fixed tunnel in zone A). Indeed, RSC is highly interconnected to visual cortex ^49-51^ and RSC cells have already been shown to fire at set distances away from single visual landmarks ^12^. To evaluate visual responses, neural activity was realigned to each zone B object’s location across laps. To determine if a cell was significantly tuned to the object, a Z-score based tuning criterion was applied to a window of activity surrounding the aligned object (see Methods; Fig. 7A&B). Similarly, to determine if each cell was significantly tuned to position, activity was aligned to each of the six potential positions into which objects could shift (within zone B) and the same tuning criterion was applied. Each cell was classified based on whether it met the tuning criterion for the position (in any of the six potential positions), object (next to any of the four objects), or both. Because a z-score cutoff of 2.33 was used (p<0.01), we anticipated a false positive rate of one percent per object or location, and six percent total given the six potential positions at which each cell’s activity was studied.

Due to overlapping alignments in the fixed environment, many cells met criterion for both object and position tuning (24.1+/−19.8% of all neurons; Fig. 7C). A smaller percentage of cells were exclusively tuned to position (3.2+/−2.8%), because they were most active in parts of the track without nearby objects. Meanwhile in the destabilized configuration, a larger proportion of cells were aligned to positions over objects (27.7+/−18.1 vs. 3.2+/−3.0%) confirming the persistence of PCCs. In the shifting configuration similar, small percentages of neurons were significantly tuned to positions (8.7+/−6.4%) as to objects (7.5+/−7.3%), and very few at both (1.1+/−0.9%). Since percentages of cells aligned to shifting objects were consistently around the false positive rate, we found no evidence that RSC cells were responsive to individual visual objects in this experiment.

### Rate remapping observed in environments with destabilized objects

When the previously stable environment was destabilized, RSC neurons continued to track the global reference frame of the familiar environment, rather than the local reference frames of individual visual objects. However, some degree of remapping was evident, and we hypothesized this could be rate remapping. Unlike prior rate remapping studies, in our study, visual cues were slightly changed on every lap. Thus, we predicted that, if rate remapping took place, there would not be a stable change in firing rates between the fixed and destabilized configurations; instead, in-field firing rates of PCCs should change on every lap of the destabilized configuration, leading to increased rate variance. Figure 7D shows how the activity of 2 example PCCs changed across the transition from fixed to destabilized objects. Note the increased standard deviation within all fields for the laps in the destabilized configuration. We calculated the peak (regardless of location) activity in each lap for all RSC PCCs when animals were running in the fixed configuration (predestabilized), and the destabilized configuration. Neurons were classified as zone A or zone B PCCs, based on the location of their average peak. Then we calculated the standard deviation of lap peaks across laps for each PCC and averaged across PCCs simultaneously recorded in each mouse (Fig 7E). The variability in zone B in the destabilized configuration was significantly greater than the variability in zone A, and in either zone pre-destabilization (Table 1). Running speed is also known to impact firing rates (see Fig S2), so to ensure this did not impact our results, we calculated the running speed at each lap’s activity peak (for each cell). We found no effect of configuration or zone (Fig 7F). We also calculated the mean in-field activity standard deviation of only fields whose peak activity moved less than +/−25 cm across the transition from fixed to destabilized, yielding similar results (supplementary Fig S4). In conclusion, once formed, RSC place fields did not show large changes in firing locations in response to shifting visual objects, but they did show rate remapping. Since rate remapping is a characteristic of hippocampal PCCs, this may be another example of information transmitted from hippocampus to RSC, but it could also be a result of non-hippocampal inputs to RSC.

## Discussion

We found evidence that position correlated activity develops in hippocampus and RSC on a similar timescale. We also showed that established position correlated cell (PCC) sequences in RSC remain consistent after the introduction of visual variability, a manipulation which otherwise disrupted field formation. This robustness of PCC sequences is a form of pattern completion/error correction which is a key property of auto-associative memories ^31,37,39^. Finally, we found that the forms of remapping in RSC are similar to those in CA1. In fact, the only differences we found between RSC and CA1 were subtle: a difference in the amount of remapping near one prominent object (the tunnel) shared across environments, a lack of experience-dependent place field expansion in RSC, and a coherence, only in CA1, of changes in spatial information across the whole environment, due to changes in only a portion of the environment.

Why would there be so much shared information across regions? According to memory index theory^52,53^, information about attributes (memory content), in neocortex, gets linked to memory indices in the hippocampus. However, there are not enough synapses between hippocampus and neocortex to permit direct transmission of sparse CA1 representations to association cortex neurons. Therefore, the index signal must first get compressed (in subiculum), and then extracted back into a sparse code in the neocortex^43,45,54-56^. We posit that PCC activity in RSC is a representation of that memory index in neocortex.

The current experiments could not establish the direction of information flow. To understand this, inactivation experiments and simultaneous recording across CA1, RSC and other regions are needed. Hippocampal lesion experiments have shown that, post-lesion, RSC place fields do not form as well in novel environments, but familiar environments retain PCCs after the lesion ^23^. This is consistent with behavioral experiments that show that over time, memories that initially depend on hippocampus are consolidated into hippocampus-independent form in neocortex ^57-59^. To further understand this process, the next step is to simultaneously monitor neural activity across regions during various stages of a memory.

We did not find robust RSC activity that tracked shifting visual objects in this experiment. This was initially surprising, as a recent study found that RSC activity primarily organizes around the location of visual cues, over the start of the trial^12^. CA1 activity has also been shown to shift in relation to one or two visual objects ^60^. This results in place cells responding to reference frames defined by independent objects as if they were separate, disconnected maps ^60,61^. There are a few possible explanations for the lack of object tuned cells in our experiment. Notably, our task had four independently shifting objects, thereby creating five independent reference frames (one for each shifting object, and one for the rest the track) that the mice would have had to learn in a few days. The mice may not have had sufficient time or capacity to learn to distinguish all these objects. Indeed, in the prior RSC study, ‘expert’ object responses were measured at 15 days. Alternatively, RSC activity may be unresponsive to such shifting objects in the first place. Lesion studies show that RSC is not necessary for object recognition, but it is for recognizing spatial relationships between the objects, and using them to navigate^62-64^. In human fMRI studies, RSC has been shown to respond strongly to stable (and therefore navigationally relevant) landmarks, compared to unstable landmarks, after some training^65^. Our findings corroborate these results, in that shifting objects with unstable spatial relationships do not elicit a consistent RSC response.

Our data are consistent with a model in which the hippocampus and retrosplenial cortex interact closely during the formation of novel environment representations. Much is still to be learned how exactly novel representations form and about the direction of information flow, but we have shown that this endeavor will require the consideration of multiple brain regions, and careful analysis of an animal’s past learning. Nevertheless, the rapid establishment of position correlated neural activity in neocortex, suggests that memory formation and its consolidation may involve the coordinated transfer of memory indices from hippocampus to neocortex.

## Methods

### EXPERIMENTAL MODEL AND SUBJECT DETAILS

#### Animals

All animal protocols and procedures were conducted with the approval of the Animal Care and Use Committee at the University of California, Irvine. Transgenic mouse lines expressing GCaMP6s in excitatory neurons were used to visualize excitatory neurons with two-photon imaging. For all hippocampal and some cortical imaging the Thy1-GCaMP6s GP4.3 line (RRID:IMSR_JAX:024275) was utilized. The remaining cortical imaging was conducted on mice resulting from a cross between the CAMK2a-tTa driver line (RRID:IMSR_JAX:007004) and a line expressing the calcium indicator GCaMP6s under the control of the tetracycline-responsive regulatory element (tetO, RRID:IMSR_JAX:024742).^66^ Mice were group-housed until the headplate implantation surgery (>P40), and housed individually after. The mice were maintained on a 12-hour light/dark cycle in the vivarium. Animals of either sex were selected for experiments. The animals were habituated to head fixation over a few days then trained to run and lick for hidden rewards in the visual VR in a series of steps that took 4-6 weeks. Mice were either water or food restricted to motivate behavior but given supplementary food or water to maintain 80% of baseline weight. Some mice were perfused after experimentation to allow for brain histology.

### METHOD DETAILS

#### Surgical procedures

Mice underwent a headplate implantation and craniotomy in either the same or separate surgeries. First, the mice were implanted with custom designed metal headplates. In preparation, connective tissue was cleared from the surface of the skull and a thin layer of Vetbond was applied. Then the headplate was affixed, at an angle parallel to the site of imaging, with black dental acrylic (Lang Dental). The second procedure was a craniotomy. For cortical imaging, a 4 mm diameter cranial window was drilled using methods described previously.^67^ The cranial window was centered either along the midline or 2 mm lateral to midline above the right hemisphere, 1.5 mm anterior to lambda. A 4mm glass coverslip (World Precision Instruments) was placed over the exposed brain and sealed with Vetbond and black dental acrylic. Occasionally bone would grow underneath the coverslip, obscuring the field of view. An additional procedure would then follow to remove the current coverslip, delicately remove the bone growth and dura with a microscapel, and replace the coverslip. For hippocampal imaging, tissue over the somatosensory cortex was aspirated and replaced with a 1.8 mm cylindrical micro-optic plastic (MOP). MOPs were formed by curing the optical polymer BIO-133^68,69^ with 395nm light in a custom-built aluminum mold. During all procedures, mice were anesthetized with isoflurane in O^2^ (2% for induction, 1-1.5% for maintenance). Carprofen (5mg/kg, s.c.) and topical lidocaine (2%, 20 mg/ml) were used as analgesics. Dexamethasone (4.8 mg/kg, i.m.) was administered 4 hours before surgery to control inflammation. Sterile eye ointment (Rugby) was used to keep the eyes hydrated during the procedure. Body temperature was stabilized to 37°C with a heating pad under control of a rectal thermoprobe. The animals recovered on a warm heating pad postsurgery and were given daily injections of Carprofen (5mg/kg, s.c.) for 3 days post-surgery.

#### Visual Virtual Reality Setup

The visual virtual reality (VR) system translated rotation of a 3D printed running wheel (37.7 cm circumference) into propulsion through a virtual circular track environment displayed on three tablets (T530NU Samsung). The animal was held by a head-fork over the wheel, and viewed the VR environment on tablets held at right angles 12 cm from the eyes (300° of visual field coverage along the azimuth). Rotations of the wheel were detected by a rotary encoder (Avago), processed by a data acquisition board (NIDAQ), and input into the computer. The animal’s licks in anticipation or during consumption of reward were detected by a capacitive lick sensor (Sparkfun), and also routed into the data acquisition board. A camera recorded the animal’s pupil (Allied Vision 1" GigE Vision). The data acquisition board output signals to open a solenoid valve for a specified amount of time, allowing water or diluted condensed milk to flow through to a reward spout placed in front of the animal’s mouth. Running speed, position along the track, licking, pupil size, and reward delivery were all recorded during the session.

Custom software written in MATLAB (Mathworks) managed the view of the VR environment. Based on movement of the wheel, the point of view along the circular track progressed forwards or backwards. The system updated at a 30 Hz refresh rate. Each VR environment was composed of a circular track with circumferences ranging from 314-502 cm. Distance in the real world was calibrated to match VR distance. Rewards were dispensed either automatically or in a lick-triggered manner at two locations within each environment (hidden reward sites). Each VR environment was also distinguished by a ceiling, floor, and wall images. Some environments had complex wall images (e.g. mountains) while others (including all the novel environments used in experiment two) all had simple repeating patterns. Visual objects consisted of 3D objects designed in Unity and positioned at various locations on either side of the circular track. The tunnel object was 30 cm long, while most other objects were 5-10 cm in width. Custom software (SmoothWalk) was used to add these attributes, move the camera (mouse’s viewpoint), and wirelessly project the environments onto the tablets.^20^

The animal began each session with the wheel blocked for 10-30 minutes. When unblocked, the animal ran for a short distance (126 cm) with the tablets blacked out, then entered into its first VR environment. The first VR environment the animal typically saw each session was the animal’s training (“familiar”) environment. The beginning of each lap was defined by the middle of the tunnel object. There were no inter-trial intervals as laps progressed continuously.

The VR environment could be programmatically changed. If the mouse was scheduled to transport into a new environment, the switch occurred when the animal was halfway through the tunnel. The animal did, therefore see the environment instantly switch through the opening in the tunnel (all the tablets flashed briefly and then the new environment appeared). VR environment and object positions were recorded and saved at the end of every session.

#### Environments

For experiment one, we designed three visually distinct environments (“Classroom, Landscape, and Sunset”), with complex backgrounds and densely populated with objects on either side of the track. Each environment had a “track” that was 10 cm wide, which was visually distinct from the rest of the floor. The mouse’s position remained within the center of that track. Two versions of each environment were created, one “small” (314 cm in circumference) and one “large” (503 cm in circumference). No mouse saw both the small and the large versions of a single environment. Each environment had two different reward locations, and some were traversed in different directions (Landscape clockwise, i.e. the curve of the track appeared rightward, and Classroom and Sunset counter-clockwise, i.e. the curve of the track appeared leftward). Mice underwent training in one of the environments (most commonly the small versions of Classroom or Sunset), and the other two environments were used for novel experiences. See Figures 1 and 2, and supplementary videos for images of the environments.

For experiments two and three, we designed five new environments (“Europa, Blue Room, Paw Room, Ornament Room, Dot Room”) each with distinctly patterned backgrounds (walls and ceiling) and distinct floors (with a different and finer pattern than the walls), and only eight objects each. The “track” on which the mouse would move within each environment was not distinguished from the rest of the floor, and was 377 cm in circumference. Each object was distinct, and located near (5-20 cm) to the track. All environments except Europa had cylindrical walls on the inside and outside of the track (inside walls ~20 cm from the track; outside walls ~100 cm from the track), such that the mouse could not see objects on the opposite side of the track. (Europa only contained outer walls). The track was divided into two equal zones, A and B, each of which contained 6 equidistant locations where objects could be located. Four objects were assigned to those 6 locations. The animal could sometimes see up to two upcoming objects. Zone B began at a different distance from the tunnel in each environment, but the tunnel object was always within zone A, and was counted as one of the four zone A objects. In the “fixed” configuration, all objects within both zones were fixed within and between sessions. Sessions in which the mice ran in the fixed configuration of one of these environments were also used in experiment one. In the “shifting” or “destabilized” configurations, the each of the four objects within zone B were randomly assigned to one of the 6 positions within the same zone at the beginning of each lap. The objects always appeared on the same side of the track and were rotated to face the same direction relative to the mouse regardless of their position. There were 360 possible configurations of the four objects. Because this object shift occurred at the start of a new lap (while the mouse was in the tunnel), the animal could not see the objects change locations from inside the tunnel. No flash occurred when objects were reconfigured within the same environment.

#### Behavior

After recovering from surgery, mice were habituated to head fixation on top of the wheel for several days then taken through a 4-6 week training procedure familiarizing them to a single VR environment (“Familiar”). We developed a multi-stage training protocol for introducing mice to head-fixation, liquid reward (water or milk) delivery through a metal spout, introduction to running in VR, and transitioning from automatically delivered rewards to operant conditioning in which the mouse had to lick in the correct location (a 15-25 cm region), in order for a reward to be delivered. The last phase allowed us to have a behavioral read-out for how well the mouse understood the location of reward delivery.

Once the animal would regularly run over 10 laps and licked in anticipation of rewards, it was moved from the training setup to an identical VR setup underneath the microscope. On the imaging setup, mice were re-habituated to the familiar environment, then introduced on separate days to a series of novel environments. The novel environments were typically re-introduced for several days, either after 10-20 laps of a previous environment or from the beginning of the new session. Mice were imaged on consecutive days with occasional breaks. Animals encountered at least one novel environment during imaging, and some encountered more. Imaging on each mouse took anywhere from 1-8 weeks, so long as the quality of the cranial window was good and animals exhibited good behavior.

To quantify licking behavior, we divided the track into 100 position bins, and determined if any licks occurred in each bin on each lap (this ensured that bursts of licks were not weighted more than single exploratory licks). The five bins prior to the beginning of each reward site were classified as anticipatory locations, and the five bins following each reward site were post-reward locations, and the rest of the track contained non-specific licking. This allowed us to analyze the behavior in the same way regardless if the rewards were delivered automatically (as was the case in most novel-environment laps), or if the mouse was required to lick (within a 10 bin zone starting at the auto-deliver location) to trigger the reward delivery (most familiar-environment laps). Lick precision was calculated as the ratio of the anticipatory lick-bins to the difference between total lick-bins and post-reward lick-bins. The chance level for this calculation is 1/9 (5 anticipatory lick-bins for each of 2 rewards divided by 100 total bins – 10 post-reward bins).

#### Two Photon Imaging

Calcium transients from GCaMP6s expressing excitatory cells were recorded using a two-photon mesoscope (Neurolabware). Excitation from a laser tuned to 920 nm (Insight X3, SpectraPhysics) was phase modulated by a pockels cell (Conoptics) then guided through table optics to a water immersion 10 mm objective (numerical aperture 0.5). Brain regions were imaged through this objective by scanning the laser bidirectionally across a specified field of view using resonant and galvanometer mirrors (Cambridge Technology) and an electrically tunable lens (Optotune). Emissions were captured and amplified by GaAsP PMT and filtered using a 510/84 nm BrightLine bandpass filter (Semrock).

The objective was lowered to focus on a depth of view between 100-300μm below the pia in cortex, and 200-300 μm below the alveus in hippocampus. From an initial 4 mm large panoramic field of view of the posterior cortex, one to three regions of interest (ROIs) were specified for fast imaging (each typically 1000 μm x 600 μm). These ROIs were placed over hippocampal CA1 or retrosplenial cortex based on vasculature, or over primary visual or somatosensory cortices based on widefield calcium imaging amplitude maps. Areas of bone growth were avoided over the course of imaging. The electrically tunable lens was used to switch depths between ROIs if necessary. ROIs were recorded at a frame rate of 6-8 Hz using the Scanbox acquisition software (Neurolabware).

### QUANTIFICATION AND STATISTICAL ANALYSIS

#### Pre-Processing

The imaging data is converted into the TIFF file format using custom software, and then run through the python Suite2P pipeline for registration and segmentation.^70^ Generally the automatic curation was sufficient, but trained undergraduate technicians manually curated cells based on morphology of the soma and plausibility of activity traces. The time varying fluorescence for each of the curated cells is taken as the average fluorescence of all pixels in each cell mask. These fluorescence traces then enter a MATLAB analysis pipeline. First, the fluorescence of each cell body is normalized by the 10 pixel wide surrounding neuropil signal $\left(F(t)=F_{\text{soma}}(t)\;\text{-}\;0.7^{*}F_{\text{neuropil}}(t)\right)$.^71^ Next, the relative fluorescence change ($\Delta F∕F_{0}$) is calculated as follows: The running baseline ($F_{0}(t)$) is calculated for each time t, by smoothening the fluorescence trace based on a running average over a time window $t_{1}$, and taking the minimum value from the smoothened trace within a time window $t_{2}$ behind the current time point, t. $t_{1}$ is set to 1 second and $t_{2}$ is set to 15 seconds. The deconvolved output from Suite2p^72^ is used for certain analyses (Spatial Information and Bayesian Decoding), but most analyses utilize the ΔF∕F.

#### Statistics

Parametric statistics were used for hypothesis testing. Linear mixed effects models were used wherever possible, to account for the hierarchical structure of the data. Signals coming from the same sessions or animals, which may influence the results but are not of importance to the experiment, were set as random effects in the models. Error bars indicate standard error of the mean.

#### Analyses

Analyses were conducted with MATLAB, Python 3 (primarily using the numpy and pandas packages for data manipulation; statsmodels, scipy, pingouin, and rpy2 for stats; and seaborn and statannotations for plotting), and GraphPad Prism.

##### Criteria for Inclusion of Data

There was a lot of variability between mice in licking behavior, training required to get to the imaging stage, and number of laps run per session, and so to ensure quality data, we removed datasets in which the mouse ran less than 10 laps in a single environment, we were not able to detect 50 or more cells, or the decoder error for the familiar environment was greater than 30 cm. In most cases, if mice did not reach the decoder error criterion within one week of imaging in the familiar environment, we stopped collecting data from that mouse, but in some cases the data was removed post-hoc. In experiment three, we used this same decoder error criterion on the pre-destabilized environment to remove both the pre-destabilized and destabilized datasets.

##### Position Binning

Certain signals, such as velocity and cell activity, were position binned and occupancy normalized. First, any time bins in which the velocity was less than 1 cm/s were eliminated, so that we did not analyze periods when the mouse was stopped or running backwards. Then we constructed an M x N matrix, where M is the total number of laps the animal ran in the environment, and N is the number of bins into which the circumference of the circular track is divided (100). The average signal at each bin is then calculated by summing over the time period the animal spent at that bin and dividing by the length of time spent at that bin. This results in a rate of activity for each entry of the position binned matrix. Note that the first and last bins of each lap are neighboring positions on the track, and are both inside the tunnel of each environment.

##### Visualization of Population Activity

To visualize position correlated sequences, lap-averaged activity of all recorded (or just spatially tuned) cells were plotted as a function of bins in the environment. Cells were ordered along the y-axis by the position of their most active bin (i.e. cells ordered based on how early along the track activity peaked). To ensure that any observed PCS was due to the cell consistently firing at that position, a different set of laps were averaged when determining the peak bin (a random subset of 5 of the final 10 laps) and for plotting (the rest of the laps in that environment). To see if the same sequence persisted across both environments A and B, the activity in environment B was plotted by sorting cells by their peak bin in A, and vice versa.

##### Classifying position-correlated cells

To identify cells that were spatially tuned in a particular environment, we used a three-step criterion. First, the position-binned activity of each cell was averaged across laps, smoothed with a Hanning window of 5 bins, and the local peaks of this average trace were found. Any peaks that were higher than 3.5 times the 50th percentile of this trace were considered candidate place fields. The boundaries of these candidate place fields were set at the closest position at which the activity dropped below the 50% percentile, or the closest trough after which the trace reached below 70% of the peak (whichever came first). Second, the peak activity on each lap within the boundary of each field was compared to the baseline activity (50th percentile minus 5th percentile of activity in all bins and all laps). The in-field activity had to be 3.5x greater than the baseline in at least of laps, or 5 laps (whichever was greater), to continue considering it as a place field. Finally, the size of the field was calculated based on the previously set boundaries, and any fields less than 20 cm, or greater than 150 cm were eliminated. Cells were considered spatially tuned if they were determined to have at least one place field. For some analyses, place fields were considered independently of the cell they belonged to, so that multiple fields belonging to one cell could be analyzed. The thresholds in this criterion (50^th^ percentile and 3.5x greater peak than baseline) had been determined by an experimenter visually inspecting resulting place fields for two hippocampal and two retrosplenial datasets (after this, the criteria were set the same for all datasets regardless of brain region). These thresholds are somewhat arbitrary, and not very stringent. Therefore, we considered all cells without using this criterion wherever possible. The percent of cells that pass the criterion in each dataset may reflect a combination of many factors, including the familiarity of the animal with the environment, the brain region, the size of the environment, imaging quality, and the number of environments visited. The last two are factors, because cells that do not have place fields do not fire often (especially in the hippocampus), and thus may be missed by our cell detection algorithm, especially when the images are dim, or not much imaging outside of the behavior period is considered. We attempted to limit this impact by imaging for 5-30 min before and after each running session (while the animal was resting on the immobile wheel in the dark), and using the whole session for cell detection. Despite this, caution should be used when interpreting the percentage of spatially tuned cells.

##### Population vector correlations

The population vector is a list of the activities of all simultaneously imaged cells in a particular time or position bin. To compare activity between laps and between environments, we calculated the activity of all cells in each position bin in a single lap, or averaged across three consecutive laps. Then we correlated the population vector in each position bin, with the same in each position bin in a different set of laps. This results in a matrix of correlations. The correlations along the diagonal represent the same position bins correlated between lap intervals. We averaged the correlations along the diagonal to get the average correlation of one lap interval with another lap interval, either in the same or in different environments. Because some environments were different sizes, we divided all environments into 100 bins, differing in size (from 3-5 cm) between environments, in order to have square population vector correlation matrices. This means that moving along the diagonal does not correspond to the same distance in different environments. However, if there was a correlation between environments at corresponding distances (instead of corresponding bins), this would be seen as an increased correlation along y=3/5x (for example), instead of the diagonal, which was not observed.

##### Spatial Information

Spatial information content (SI) quantifies the information available to locate the animal based on neuronal firing rate.^73^ We calculated the SI for each cell based on the lap-averaged deconvolved signal (because deconvolved signal, unlike ΔF∕F, cannot be negative at any point) based on this formula:

$$SI=\sum_{i=1}^{N}p_{i}\frac{f_{i}}{f}\;log_{2}\;\frac{f_{i}}{f}$$

N stands for the total number of bins, $p_{i}$ is the probability of occupying the $i^{\text{th}}$ bin, $f_{i}$ is the deconvolved activity in the $i^{\text{th}}$ bin, and f is the activity averaged across all $f_{i}$ bins. The measure is quantified in bits. The deconvolved activity is shuffled 100 times (each lap was circularly shifted by a random integer) to also obtain a null distribution of spatial information scores.

##### Sparsity

We measure lifetime sparsity of each neuron to determine if the cell is narrowly or broadly active across position bins, using the following formula:

$$sparsity=\frac{(\sum_{i=1}^{N}p_{i}f_{i}{)}^{2}}{\sum_{i=1}^{N}p_{i}f_{i}^{\;2}}$$

The variables are the same as those used in SI. Sparsity values closer to 0 indicate sparser representations, while values close to 1 indicate broad activity across many position bins.

##### Bayesian Decoding

A Bayesian decoder was used to estimate the animal’s position based on neural population activity.^74^ At every time point, the posterior probability of being at a particular location on the track, given the neural population activity, was calculated based on the prior probability of being at a particular position multiplied by the likelihood of this neural activity being produced at that position, divided by a normalization term. The model is trained with leave one out cross-validation; so time points corresponding to when the animal is on a particular lap are decoded based on activity on all other time points (corresponding to all the other laps). The formula for this is expressed as follows:

$$P(x|n)=C\left(\prod_{i=1}^{N}f_{i}(x{)}^{n_{i}}\right)\text{exp}\left(-\tau \sum_{i=1}^{N}f_{i}(x)\right)$$

$f_{i}$ is the mean deconvolved, Gaussian-smoothed fluorescence trace over position x and $n_{i}$ is the time course vector of the $i^{\text{th}}$ neuron within a time bin of length τ, which we empirically optimized to be 3 seconds. N corresponds to the number of neurons, and constant C normalizes the probability distribution to sum to 1 across all positions. A decoded position was defined as the position with the highest probability for any given time bin, and the absolute value of the difference between true position and decoded position was defined as the Bayesian decoding error.

##### Tuning to Position or Object Reference Frames

In analysis for object tuning, position binned neural activity on each session is aligned to three different reference frames over a window spanning 9 cm prior to the object (3 position bins) and 3 cm past (1 position bin). First, activity is aligned to the actual position of each of the four objects in zone B. This position varies randomly from lap to lap in the shifting configuration but stays in one location in the fixed configuration. Second, activity is aligned to each of the six potential locations where objects could be. This is similar to the object alignment in the fixed configuration, but includes two spots not near any objects. The potential positions are at least 1/12 of a track length away from the other positions. And third, to see what could occur by chance, activity is aligned to a set of possible locations randomly chosen from every lap. This randomization procedure is repeated 1000 times to obtain a shuffle distribution for each neuron.

Cells were deemed to be significantly tuned at any object or position: 1) if the lower bound of the trial-averaged activity (mean - SEM) was significantly greater than the shuffled distribution (97.5 percentile) at any position bin within the window; and 2) if the trial and position-binned average activity within the window has a z-score over 2.3263 (p=0.01) relative to the trial and position-binned averages of the shuffled distribution. This double criterion ensures that tuned cells are significantly active at the same relative distance away from a particular position or object on most trials.

##### Activity variability across laps

In order to quantify possible rate remapping in destabilized environments, we measured the standard deviation of peak activity and average in-field activity between laps. Since there were 360 possible configurations of the four objects in zone B, no configuration occurred more than once in a session, and we could not assess rate remapping as traditionally done by comparing firing rates between configurations. However, if the rate changes occurred in every configuration, then the variability of firing rates should be greater in the shifting configuration compared to the stable configuration. The activity of each cell was first z-scored, and then we calculated the average in-field activity and the peak activity in each lap for spatially tuned cells. To find the peak activity, the z-scored activity for each cell was first convolved with a Hanning window of 5 bins, to get a less noisy result. Cells were classified as having a field in zone A or zone B, depending on in which zone the peak firing rate (across laps) occurred, and zone A and zone B cells were considered separately and compared. We also compared cells in the stable version of the environment with cells in the shifting version of the same environment, using a two-way repeated measures ANOVA. In order to determine if running speed was a factor in firing rate changes, we also found the mouse’s velocity in the position bin in which each cell’s lap peak occurred, and ran the same analysis with these velocities.

## Figures and Tables

**Table 1:** Summary of all statistics tests

Figure	Variable	N	Test	Null hypothesis	Results	Statistic
2D	Location of field in familiar vs. location in novel	RSC: 703 fields	Correlation	Locations are uncorrelated across environments	Reject null	r=0.24; p<0.001
		RSC: 443 fields not near tunnel	Correlation	Locations of fields away from the tunnel are uncorrelated	Accept null	r=0.09; p=0.053
2E	Percent of RSC cells with fields in both environments	RSC: 18 sessions	T-test	Percent of cells is same as mathematically expected value	2.32% higher than expected; reject null	t=6.80; p<0.001
				Percent of cells away from tunnel is same as expected value	1.70% higher than expected; reject null	t=5.96; p<0.001
2G	Location of field in familiar vs. location in novel	CA1: 574 fields	Correlation	Locations are uncorrelated across environments	Accept null	r=0.01; p=0.78
2H	Percent of CA1 cells with fields in both environments	CA1: 10 sessions	T-test	Percent of cells is same as mathematically expected value	0.76% higher than expected; accept null	t=1.71; p=0.121
3C	PV correlation with last 3 laps in familiar	RSC: 18 sessions	One way repeated measures ANOVA	There is no difference across laps	Reject Null	F=140; p<0.0001
		CA1: 10 sessions	One way repeated measures ANOVA	There is no difference across laps	Reject Null	F=44; p<0.0001
		18 vs. 10 sessions	Two way repeated measures ANOVA	There is no interaction between lap and brain region	Accept Null	F=0.69; p=0.41
3D	PV correlation with late laps in novel	RSC: 18*10 laps	Linear regression	There is no relationship between PV correlation and lap	R^2^ = 0.15	p<0.0001
		CA1: 10*10 laps			R^2^ = 0.11	p<0.001
		180 vs. 100	Compare linear regression parameters	RSC and CA1 slopes are equal RSC and CA1 intercepts are equal	Accept null Reject null	F(1,276)=0.44; p=0.51 F(1,277)=19; p<0.0001
				RSC and CA1 intercepts are equal	Reject null	F(1,277)=19; p<0.0001
3E	PV correlation with 7 laps away	RSC: 18*10 laps	Linear regression	There is no relationship between PV correlation and lap	R^2^ = 0.065	p<0.001
		CA1: 10*10 laps			R^2^ = 0.013	p=0.29
		179 vs. 93	Compare linear regression parameters	RSC and CA1 slopes are equal	Accept null	F(1,268)=0.76; p=0.38
				RSC and CA1 intercepts are equal	Reject null	F(1,269)=34; p<0.0001
3	Decoder error in best 5 laps	RSC: 18 sessions	One-tailed paired T-test	Familiar and novel environments are the same	Familiar 5.2 +/− 2.3 cm; Novel 14.2 +/− 12.8 cm; Reject null	p<0.01
		CA1: 10 sessions			Familiar 3.8 +/− 1.2; Novel 11.2 +/− 10.5; Reject null	p<0.05
3F	Amount of time decoder error is >20 cm	RSC: 18*15 laps	Linear regression	Slope is not different from zero	Y= −0.017X+0.43; R^2^ = 0.12	F(1,268)=37; p<0.0001
		CA1: 10*15 laps			Y= −0.16X+0.38; R^2^ = 0.082	F(1,148)=13; p<0.001
		270 vs. 150	Fit an exponential decay with offset	One curve fits both datasets	Accept null; best fit curve: Y=0.41*exp^−0.34X^ + 0.21	F(3,414)=2.1; p=0.093
				Line is a better fit than exponential	Reject null	RSC: F=8.5; p<0.01 CA1: F=8.3; p<0.01
4B	Lap averaged Z-scored activity	RSC: 1129 cells	T-test	There is no difference in activity between the lap prior to and after the transition for cells with fields in both environments.	Reject null	t=4.17, p<0.001
		CA1: 1138 cells			Reject null	t=7.10, p<0.001
4D	In-out activity ratio	RSC: 18*15 laps	Linear regression	Slope is not different from zero	Y = 0.014X+1.22; R^2^ = 0.13	F(1,268)=39; p<0.0001
		CA1: 10*15 laps			Y = 0.032X+1.11; R^2^ = 0.11	F(1,148)=19; p<0.0001
		270 vs. 150	Compare linear regression parameters	RSC and CA1 slopes are equal	Reject null	F(1,416)=7.9; p<0.01
4E	COM shift in familiar	RSC: 18 sessions	One way repeated measures ANOVA	There is no difference across laps	Accept null	F=1.8; p=0.20
		CA1: 10 sessions			Reject null	F=20.4; p<0.01
		18 vs. 10	Two way repeated measures ANOVA	There is no difference across laps	Reject null	F=8.8; p<0.01
				There is no interaction between laps and brain region	Reject null	F=4.6; p<0.05
	COM shift in novel	RSC: 12 sessions with >12% PCCs	One way repeated measures ANOVA	There is no difference across laps	Accept null	F=1.3; p=0.28
		CA1: 5 sessions with >12% PCCs			Trend toward reject null	F=7.2; p=0.055
4F	Place field size in familiar	RSC: 18 sessions	One way repeated measures ANOVA	There is no difference across laps	Accept null	F=3.2; p=0.091
		CA1: 10 sessions			Reject null	F=6.9; p<0.05
		18 vs. 10	Two way repeated measures ANOVA	There is no difference across laps	Accept null	F=0.69; p=0.41
				There is no interaction between laps and brain region	Reject null	F=7.2; p<0.05
	Place field size in novel	RSC: 12 sessions with >12% PCCs	One way repeated measures ANOVA	There is no difference across laps	Accept null	F=1.0; p=0.34
		CA1: 5 sessions with >12% PCCs			Reject null	F=14.7; p<0.05
		12 vs. 5	Two way repeated measures ANOVA	There is no difference across laps	Reject null	F=8.5; p<0.01
				There is no interaction between laps and brain region	Reject null	F=4.6; p<0.05
5C	Percent PCCs in Zone B	RSC: 10 sessions fixed; 8 sessions shifting	Kolmogorov-Smirnov test	The same percent of PCCs is located in zone B in the shifting configuration as in the fixed configuration	Fixed: 34.5+/−14.3%; Shifting: 8.73+/−7.14%; Reject null	p<0.01
		CA1: 6 sessions fixed; 7 sessions shifting			Fixed: 23.9+/−11.1%; Shifting: 16.9+/−10.5%; Accept null	p=0.212
	Spatial information content	RSC: 10 sessions fixed; 9 sessions shifting	Linear mixed effects model	There is no influence of configuration or zone or their interaction	Reject null	Configuration: p=0.899; Zone: p=0.207; Interaction: p<0.01
				There is no difference between fixed and shifting in Zone A	Accept null	p=0.663
				There is no difference between fixed and shifting in Zone B	Reject null	p<0.0001
		CA1: 6 sessions fixed; 7 sessions shifting		There is no influence of configuration or zone or their interaction	Reject null	Configuration: p<0.001; Zone: p<0.05; Interaction: p=0.81
				There is no difference between fixed and shifting in Zone A	Reject null	p<0.01
				There is no difference between fixed and shifting in Zone B	Reject null	p<0.0001
5D	Decoding error	RSC: 10 sessions fixed; 9 sessions shifting	Linear mixed effects model	There is no influence of configuration or zone or their interaction	Reject null	Configuration: p=0.982 Zone: p=0.622 Interaction: p<0.001
				There is no difference between fixed and shifting in Zone A	Mean = 39.17 vs. 39.02 cm; Accept null	p=0.982
				There is no difference between fixed and shifting in Zone B	Mean = 37.17 vs. 64.62 cm; Reject null	p<0.001
		CA1: 6 sessions fixed; 7 sessions shifting		There is no influence of configuration or zone or their interaction	Reject null	Configuration: p=0.076 Zone: p=0.833 Interaction: p=0.001
				There is no difference between fixed and shifting in Zone A	Mean = 34 vs. 46 cm; Accept null	p=0.177
				There is no difference between fixed and shifting in Zone B	Mean = 33 vs. 68 cm; Reject null	p<0.001
6B	Percent cells which are PCCs	RSC: 6 sessions pre-destabilized; 6 sessions destabilized	Wilcoxon matched-pairs signed rank test	The same percent of cells qualify as PCCs in the pre-destabilized session as in the destabilized session.	Pre-destabilized: 25.6+/17.2%; Destabilized: 24.8+/−18.7%; Accept null	p=0.844
		CA1: 4 sessions pre-destabilized; 4 sessions destabilized			Pre-destabilized: 40.0+/9.2%; Destabilized: 32.4+/−9.2%; Accept null	p=0.125
	Percent PCCs in zone B	RSC: 6 sessions pre-destabilized; 6 sessions destabilized	Wilcoxon matched-pairs signed rank test	The same percent of PCCs is located in zone B in the pre-destabilized session as in the destabilized session	Pre-destabilized: 34.5+/15.8%; Destabilized: 27.5+/−20.5%; Accept null	p=0.313
		CA1: 4 sessions pre-destabilized; 4 sessions destabilized			Pre-destabilized: 38.7+/8.9%; Destabilized: 32.7+/−18.6%; Accept null	p=0.875
6C	Decoding error	RSC: 6 sessions pre-destabilized and destabilized; 9 sessions shifting day 1	Linear mixed effects model and Tukey’s Post-Hoc test	There is no influence of configuration or zone or their interaction	Reject null	Configuration: p=0.005 Zone: p=0.020 Interaction: p<0.001
				There is no difference between configurations in Zone A	Pre-destabilized (17 cm) vs destabilized (20 cm); Accept null	p=0.590
					Shifting day 1 (39 cm) vs destabilized (20 cm); Reject null	p<0.001
					Pre-destabilized (17 cm) vs shifting day 1 (39 cm); Reject null	p<0.001
				There is no difference between configurations in Zone B	Pre-destabilized (19 cm) vs destabilized (31 cm); Reject null	p=0.042
					Shifting day 1 (65 cm) vs destabilized (31 cm); Reject null	p<0.001
					Pre-destabilized (19 cm) vs shifting day 1 (65 cm); Reject null	p<0.001
		CA1: 4 sessions pre-destabilized and destabilized; 7 sessions shifting day 1		There is no influence of configuration or zone or their interaction	Reject null	Configuration: p<0.001 Zone: p=0.117 Interaction: p=0.134
				There is no difference between configurations	Pre-destabilized (9 cm) vs destabilized (19 cm); Accept null	p=0.143
					Shifting day 1 (33 cm) vs destabilized (19 cm); Reject null	p=0.038
					Pre-destabilized (9 cm) vs shifting day 1 (33 cm); Reject null	p<0.001
7E	Variability of lap peak	RSC: 6 sessions	Two-way ANOVA	There is no influence of configuration or zone or their interaction	Reject null	Configuration: p<0.01 Zone: p<0.001 Interaction: p<0.01
			Multiple comparisons: compare each group mean with every other group mean	Zone A: pre-destabilized vs. destabilized	Accept null	p=0.86
				Pre-destabilized: zone A vs. zone B	Accept null	p=0.99
				Pre-destabilized zone A vs. destabilized zone B	Reject null	p<0.01
				Destabilized zone A vs. pre-destabilized zone B	Accept null	p=0.98
				Destabilized: zone A vs. zone B	Reject null	p<0.05
				Zone B: pre-destabilized vs. destabilized	Reject null	p<0.05
7F	Variability of running speed at leap peaks	RSC: 6 sessions	Two-way ANOVA	There is no influence of configuration or zone or their interaction	Accept null	Configuration: p=0.46 Zone: p=0.91 Interaction: p=0.42
